# A Rare Case of Pott's Puffy Tumor in an Adult With Paranasal Osteoma and Pachymeningitis

**DOI:** 10.7759/cureus.82120

**Published:** 2025-04-11

**Authors:** Brian G Nudelman, Jeffrey Valencia Uribe, Nicole Nudelman, Ann-Katrin Valencia, Kenneth Poon

**Affiliations:** 1 Internal Medicine, Memorial Healthcare System, Pembroke Pines, USA; 2 College of Medicine, Kansas City University, Kansas City, USA; 3 College of Medicine, Florida International University, Herbert Wertheim College of Medicine, Miami, USA; 4 Infectious Disease, Memorial Healthcare System, Pembroke Pines, USA

**Keywords:** frontal osteomyelitis, orbital cellulitis, pachymeningitis, pott's puffy tumor, subperiosteal abscess

## Abstract

Pott's puffy tumor (PPT) is a rare but potentially life-threatening complication of frontal sinusitis, most commonly seen in children and adolescents. We report an unusual case of PPT in a 63-year-old man with a history of intranasal cocaine use, complicated by pachymeningitis. The patient presented with severe right eye pain, periorbital swelling, erythema, and purulent discharge. Initial imaging revealed right orbital cellulitis and erosive paranasal sinus disease, with a suspected frontal subgaleal abscess. Brain MRI confirmed the diagnosis of PPT, demonstrating acute-on-chronic pansinusitis, frontal bone osteomyelitis, subperiosteal phlegmon, and associated pachymeningitis.

The patient was managed with broad-spectrum intravenous antibiotics, with surgical intervention considered. Notably, he demonstrated clinical improvement with medical management alone, including the resolution of periorbital edema. This case underscores the evolving epidemiology of PPT in the adult population and highlights the role of risk factors such as cocaine use and sinus osteoma. It also emphasizes the critical importance of early recognition and a multidisciplinary approach in managing PPT to prevent serious intracranial complications. Despite the anatomical complexity, conservative medical therapy can be effective in selected cases without immediate surgical indications.

## Introduction

Pott's puffy tumor (PPT) is an uncommon yet potentially life-threatening complication of frontal sinusitis, typically observed in children and adolescents and only rarely reported in adults [1]. However, an increasing number of adult cases have been documented over the past two decades. PPT is characterized by the formation of a subperiosteal abscess over the frontal bone, often accompanied by frontal scalp swelling (79.1%), headache (56.7%), fever (35.5%), periorbital edema or erythema (23.4%), and rhinorrhea (19.3%). Orbital involvement, although less frequent, has been reported in approximately 24-29% of cases [2,3]. Intracranial complications are a serious concern, with reported incidences ranging from 30% to 85%. Nonetheless, neurological symptoms, such as seizures, meningeal signs, and focal neurological deficits, remain relatively uncommon, occurring in approximately 10.6% of patients [3,4]. Notably, up to 24.7% of adult cases may present without overt clinical manifestations [3].

It has been hypothesized that PPT results from an anatomically underdeveloped frontal sinus and increased blood flow through the diploic veins of the frontal bone, predisposing to infection [5]. Bacterial overgrowth within the frontal sinus can exacerbate mucosal venous congestion and promote septic thrombophlebitis, ultimately leading to local osteomyelitis. The microbial profile is often polymicrobial with an anaerobic predominance. Commonly isolated pathogens include *Streptococcus *species (35.5%), *Staphylococcus *species (21.2%), *Fusobacterium *(5.3%), *Pseudomonas *(3.4%), and *Prevotella *(2.2%) [4,5]. Recognized comorbidities that increase susceptibility to PPT include head trauma, prior sinus or neurosurgical procedures, immunosuppression, diabetes mellitus, and intranasal cocaine use. We present a case of PPT in a 63-year-old man with a significant history of cocaine use, complicated by pachymeningitis.

## Case presentation

**Figure 1:**
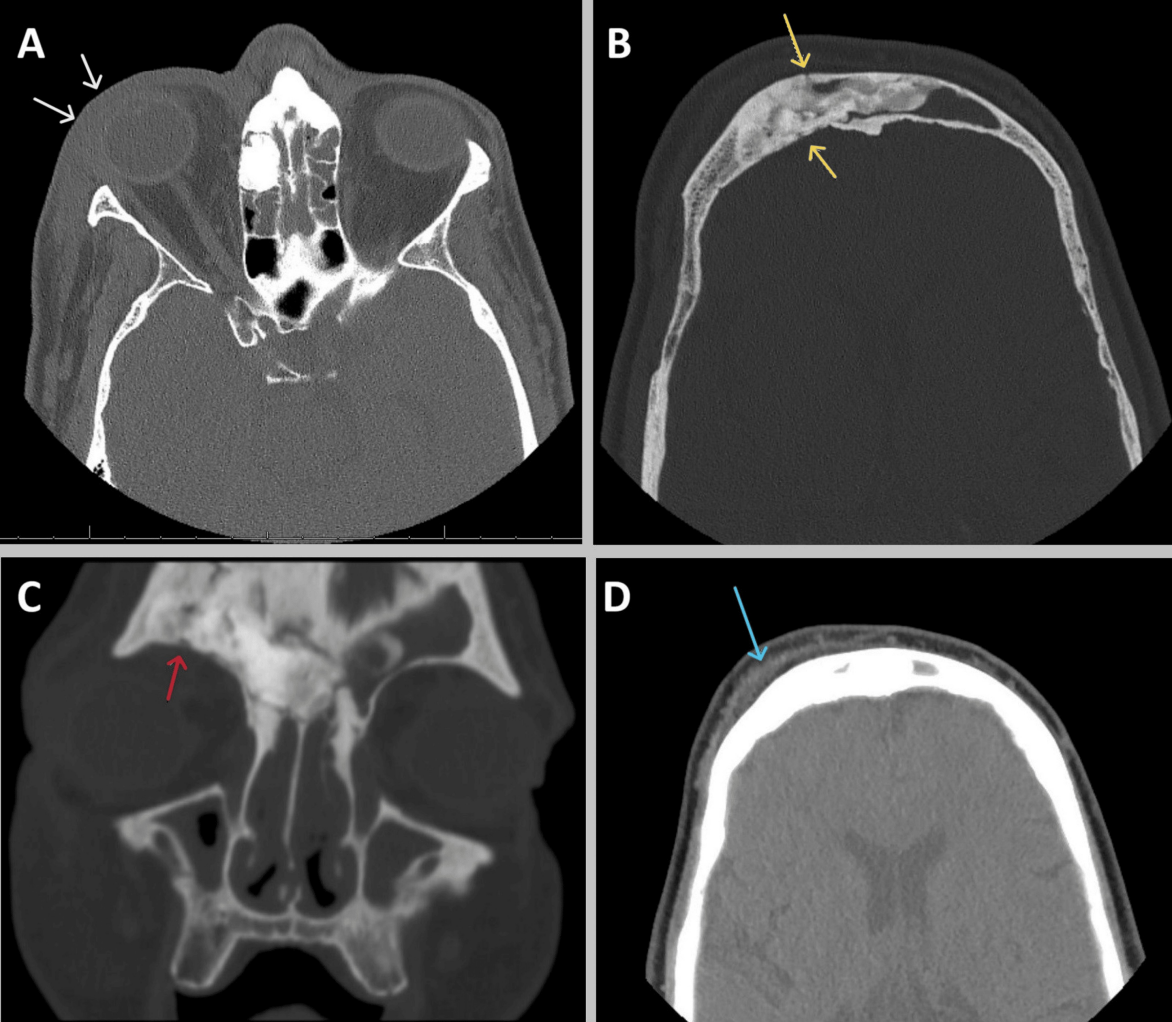
CT of the orbits without IV contrast CT of the orbits without IV contrast showed post-septal fat stranding and edema involving the superior extraconal aspect of the orbit (A: white arrows) compatible with right orbital/post-septal cellulitis and opacification of the frontal sinuses (B: yellow arrows) consistent with severe paranasal sinus disease. There were subtle erosive changes of the right frontal sinus anterior and posterior walls, as well as the floor of the right frontal sinus (right orbital roof; C: red arrow), with possible frontal subgaleal collection (D: blue arrow) secondary to sinus infection.

We present the case of a 63-year-old man with a past medical history of prediabetes, hypertension, and daily nasal inhalation of cocaine and crushed oxycodone. He presented to the emergency department with three days of severe right eye pain, swelling, and redness. Upon admission, the patient's vital signs were as follows: blood pressure of 140/88 mmHg, temperature of 36.7°C, heart rate of 84 bpm, and respiratory rate of 15 breaths per minute. Physical examination revealed severe right forehead and periorbital edema, with associated right eye chemosis, conjunctival injection, and purulent nasal discharge. The ophthalmoscopic examination revealed no damage to the retina, conjunctiva, or optic nerve. Lab results showed the following: neutrophilic leukocytosis 11.4×10^3^/µL (reference range: 4.0-11.0×10^3^/µL), elevated inflammatory markers C-reactive protein 7.9 mg/dL (reference range: <0.5 mg/dL) and erythrocyte sedimentation rate 43 mm/h (reference range: <20 mm/h), lactic acid within normal limits (reference range: 0.5-2.2 mmol/L), and hemoglobin A1c 5.7% (reference range: <5.7%). Blood and urine cultures were negative. A CT scan of the orbits revealed right orbital cellulitis with severe erosive paranasal sinus disease and a possible right frontal subgaleal abscess (Figure 1). 

**Figure 2:**
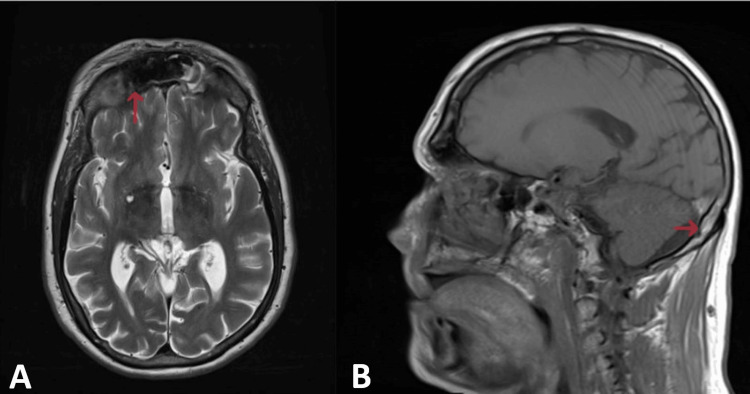
MRI of the brain with and without IV contrast MRI of the brain with and without IV contrast showed acute-on-chronic pansinusitis with associated frontal bone osteomyelitis (A: red arrow on axial view) and subperiosteal phlegmon suggestive of Pott's puffy tumor with complications of pachymeningitis (B: red arrows on sagittal view). Additional findings of periorbital cellulitis were also found. No cerebritis, brain abscess, acute infarct, or hemorrhage was noted.

An MRI of the brain showed pansinusitis, with associated frontal bone osteomyelitis, consistent with PPT (Figure 2). The MRI also revealed subperiosteal phlegmon, periorbital cellulitis, and pachymeningitis.

The patient was started on IV cefepime, metronidazole, vancomycin, and ciprofloxacin eye drops for post-septal cellulitis and frontal bone osteomyelitis. Dexamethasone was also initiated to prevent damage to the optic structures. Surgical intervention was determined to have an unfavorable risk/benefit ratio because of the complexity of navigating the osteoma.

**Figure 3:**
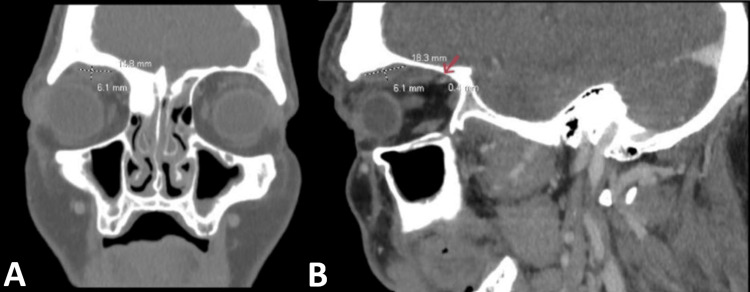
CT of the sinus with IV contrast CT of the sinus with IV contrast showed large ossified lesions compatible with an osteoma nearly completely occluding the right frontal sinus and extending into the left frontal sinus (A: coronal view; B: sagittal view). The subperiosteal abscess (marked by dash lines) in the right orbital wall measuring 1.83×1.48×0.6 cm with associated mild exophthalmos was stable compared to the previous CT of the orbits. The right frontal sinus subgaleal abscess/phlegmon (red arrow) has improved in thickness from 0.6 cm to 0.4 cm compared to the last CT of the orbits.

The patient demonstrated significant improvement in periorbital swelling by day 3 of antibiotic therapy. In light of this progress, cefepime was discontinued on day 4 and replaced with ceftriaxone. However, by day 7, the patient experienced worsening periorbital edema, marked proptosis, and copious bloody mucosal discharge. A contrast-enhanced CT scan of the sinuses was performed, revealing a large right frontal calcified osteoma, a stable subperiosteal abscess, and a reduction in the size of the previously noted subgaleal abscess/phlegmon on MRI (Figure 3). Ceftriaxone, vancomycin, and metronidazole were continued, leading to the gradual resolution of the swelling. The patient was ultimately discharged to a skilled nursing facility to complete the remaining six weeks of IV antibiotic therapy, given concerns related to the risk of IV drug use.

## Discussion

PPT is typically seen in the pediatric population; however, in recent years, there's been a rise in its incidence in adults, with a prevalence of 47% in the pediatric and 53% in the adult population [3]. In both pediatric and adult populations, the primary cause for PPT is sinusitis infection, which occurs more often in adolescents due to the incomplete development of the frontal sinuses and increased diploic venous blood flow, leading to an increased risk of translocation of infection to the surrounding frontal bone [6]. One possible explanation for the decreased incidence is the advancement of broad-spectrum antibiotics. However, other risk factors are more commonly seen in adults, such as odontogenic disease and diabetes, which could explain the increased prevalence in adults. This patient has multiple risk factors, including intranasal drug use, which has been associated with up to 11.8% of adult cases [3].

The chronic use of cocaine is associated with sinus inflammatory changes that lead to chronic destruction of the osteocartilaginous structure of the sinuses and mucosal membrane, increasing the risk of recurrent sinus infection [7]. This also propagates local vascular wall changes, increasing the risk of diploic septic thrombophlebitis and spread to the frontal skull, leading to osteomyelitis. Another risk factor is the osteoma seen in the CT of the sinus (Figure 3), showing significant occlusion of the right frontal sinus. This has been previously reported in a pediatric case of PPT secondary to paranasal sinus osteoma, likely contributing to the distortion of the normal sinus anatomy and increasing the risk of recurrent sinus infection [8].

The patient's presentation of right forehead swelling with associated periorbital swelling is part of the classical findings of PPT, which guided our clinical diagnosis [3,9]. Initial CT of the orbits without IV contrast, performed due to the significant periorbital edema, showed findings of erosive paranasal sinus disease with right orbital cellulitis and a possible right frontal subgaleal abscess suggestive of sinusitis origin (Figure 1). MRI of the brain is the study of choice to assess intracranial involvement and should not be delayed if there are signs of increased intracranial pressure; however, neurological deficits are not always evident on initial presentation [9,10]. In this case, due to significant orbital involvement consistent with periorbital cellulitis, an MRI with and without IV contrast was performed (Figure 2). The imaging revealed acute-on-chronic pansinusitis with associated frontal bone osteomyelitis and a subperiosteal phlegmon, confirming the diagnosis of PPT with a complication of pachymeningitis. Additional recognized complications of PPT include meningitis; epidural, subdural, or intraparenchymal abscess or empyema; cavernous sinus thrombosis; dural venous sinus thrombosis; orbital cellulitis; and orbital abscess [4,11].

The unique aspect of this case is that the extracranial complication of periorbital cellulitis was the primary clinical feature that co-occurred with the intracranial complication of asymptomatic pachymeningitis, which has been seen in some cases of pediatric PPT [3]. Therefore, clinicians should maintain a low threshold for suspicion of intracranial involvement when PPT with orbital involvement is suspected, even without neurological deficits or meningeal signs.

Successful PPT treatment usually requires a combination of surgical drainage and debridement of infected material along with appropriate antimicrobial therapy. Disease progression occurs significantly more often with medical treatment alone than with a combined medical and surgical approach (33% vs. 13.2%; p=0.022) [3]. Some data suggest that the incidence of intracranial complications is not affected by the timing of surgical intervention (whether before or after completing antibiotics) [12]. Broad-spectrum antibiotics should be aimed at upper airway organisms common in acute bacterial rhinosinusitis (*Streptococcus pneumoniae*, *Streptococcus pyogenes*, *Staphylococcus aureus*, *Haemophilus influenzae*, *Fusobacterium*, *Peptostreptococcus*). Published cases of successful treatment have reported a range of one week to seven months of treatment duration. We recommend 6-8 weeks of antimicrobial therapy after source control for the adequate treatment of osteomyelitis.

Our treatment regimen included ceftriaxone, vancomycin, and metronidazole for coverage of the two most common organisms, *Streptococcus* and *Staphylococcus*, which make up approximately 50% of cases, as well as anaerobic coverage [3,13]. It is important to note that antibiotic treatment should be continued even if no organism is identified, as sterile cultures can occur in about 14% of cases. Treatment length is usually 6-8 weeks after source control to treat osteomyelitis [6]. In a retrospective study of 32 patients with PPT, the timing of surgical intervention, whether before or after completing antibiotics, did not correlate with the incidence of intracranial complications [12]. In our case, surgical intervention was not immediately necessary. Furthermore, due to the complex anatomy in the setting of the large osteoma and significant surrounding inflammation, there could be an increased risk of intraoperative complications. The CT of the sinus (Figure 3) also showed that the subgaleal phlegmon or abscess had decreased in thickness from 0.6 cm to 0.4 cm, showing improvement with antibiotic treatment.

## Conclusions

PPT is an uncommon complication of frontal sinusitis mostly observed in children due to incomplete frontal sinus formation. However, adult cases are increasingly reported, especially in individuals with intranasal drug use or anatomical sinus irregularities. This case emphasizes the importance of evaluating for intracranial or orbital involvement even when symptoms of intracranial disease are absent. The successful management of PPT often requires a combination of surgical intervention and medical treatment, though, as demonstrated in this case, medical therapy alone can sometimes be effective, particularly when surgery carries significant risks.

With the increasing incidence of PPT in adults, it is crucial to raise awareness among clinicians, especially when treating patients with relevant risk factors. Early and accurate diagnosis, supported by appropriate imaging and specialist consultation, is important to prevent severe complications. The combination of broad-spectrum antibiotics and close monitoring can lead to favorable outcomes, but clinicians should closely monitor patients for signs that might necessitate surgical intervention. A multidisciplinary approach is essential to ensuring the best possible outcome for patients with this complex and potentially life-threatening condition.
